# Enhanced Recovery After Surgery Protocols in One- or Two-Level Posterior Lumbar Fusion: Improving Postoperative Outcomes

**DOI:** 10.3390/jcm13206285

**Published:** 2024-10-21

**Authors:** Ji Uk Choi, Tae-Hong Kee, Dong-Ho Lee, Chang Ju Hwang, Sehan Park, Jae Hwan Cho

**Affiliations:** Department of Orthopedic Surgery, Asan Medical Center, University of Ulsan College of Medicine 88, Olympic-ro 43-gil, Songpa-gu, Seoul 05505, Republic of Korea; fairytales2@naver.com (J.U.C.); taehongkee@gmail.com (T.-H.K.); osdlee@gmail.com (D.-H.L.); baski47@gmail.com (C.J.H.); birdone86@gmail.com (S.P.)

**Keywords:** enhanced recovery after surgery, ERAS, lumbar spinal fusion, opioid reduction, antiemetic use, multimodal analgesia, postoperative outcomes, postoperative pain management, functional recovery

## Abstract

**Background/Objectives:** Enhanced recovery after surgery (ERAS) protocols optimize perioperative care and improve recovery. This study evaluated the effectiveness of ERAS in one- or two-level posterior lumbar fusion surgeries, focusing on perioperative medication use, pain management, and functional outcomes. **Methods:** Eighty-eight patients undergoing lumbar fusion surgery between March 2021 and February 2022 were allocated into pre-ERAS (*n* = 41) and post-ERAS (n = 47) groups. Outcomes included opioid and antiemetic consumption, pain scores (numerical rating scale (NRS)), functional recovery (Oswestry Disability Index (ODI) and EuroQol 5 Dimension (EQ-5D)), and complication rates. Pain was assessed daily for the first four postoperative days and at 6 months. Linear Mixed Effects Model analysis evaluated pain trajectories. **Results:** The post-ERAS group showed significantly lower opioid (*p* = 0.005) and antiemetic (*p* < 0.001) use. No significant differences were observed in NRS pain scores in the first 4 postoperative days. At 6 months, the post-ERAS group reported significantly lower leg pain (*p* = 0.002). The time:group interaction was not significant for back (*p* = 0.848) or leg (*p* = 0.503) pain. Functional outcomes at 6 months, particularly ODI and EQ-5D scores, showed significant improvement in the post-ERAS group. Complication rates were lower in the post-ERAS group (4.3% vs. 19.5%, *p* = 0.024), while hospital stay and fusion rates remained similar. **Conclusions:** The ERAS protocol significantly reduced opioid and antiemetic use, improved long-term pain management and functional recovery, and lowered complication rates in lumbar fusion patients. These findings support the implementation of ERAS protocols in spinal surgery, emphasizing their role in enhancing postoperative care.

## 1. Introduction

Enhanced Recovery After Surgery (ERAS) protocols are widely accepted across various surgical specialties as a method to optimize postoperative outcomes, reduce complication rates, and shorten the length of hospital stay [1]. This multimodal approach, initially introduced as “Fast-Track Surgery” by Henrik Kehlet in the 1990s to perioperative care, has evolved significantly [2]. Since its introduction in 2010, the ERAS Society has developed evidence-based guidelines tailored to numerous surgical procedures, with an emphasis on the importance of pre-operative education, early mobilization, multimodal analgesia, and enhanced nutritional support, which improve surgical outcomes and enhance patient experiences (http://www.erassociety.org, accessed on 1 March 2024) [1,3,4,5].

The integration of ERAS protocols into spine surgery, particularly lumbar fusion procedures, is a recent development [2]. Lumbar fusion remains a painful and complex surgical procedure despite significant advancements in spinal surgery techniques and a deeper understanding of spinal biomechanics [6,7]. Furthermore, it is frequently associated with considerable postoperative complications and a prolonged recovery period [8,9]. The increasing complexity of these procedures, combined with an aging population and the increasing demand for surgical interventions, underscores the critical need to implement standardized, evidence-based perioperative care protocols to improve recovery outcomes and reduce healthcare costs [2,10,11].

ERAS protocols can effectively reduce postoperative pain, decrease opioid consumption, and shorten the length of hospital stays following lumbar fusion surgery [2,12]. However, most previous studies have focused on broad patient populations or varied surgical procedures. Thus, the specific application of ERAS protocols in more narrowly defined contexts, such as one- or two-level posterior lumbar spinal fusion surgeries (which are commonly performed in Korea), remains unclear [13,14]. This gap in the literature underscores the necessity for conducting more focused investigations to determine the efficacy and safety of ERAS protocols in these specific surgical settings.

Therefore, this study aims to provide a detailed evaluation of the clinical effectiveness of ERAS protocols specifically in one- or two-level posterior lumbar spinal fusion surgery. This study strives to generate valuable data that contributes to the existing body of literature on ERAS by examining the postoperative outcomes. These findings will support the integration of ERAS protocols in spinal fusion surgery with a more targeted and evidence-based approach.

## 2. Materials and Methods

### 2.1. Study Design and Patient Selection

**Figure 1:**
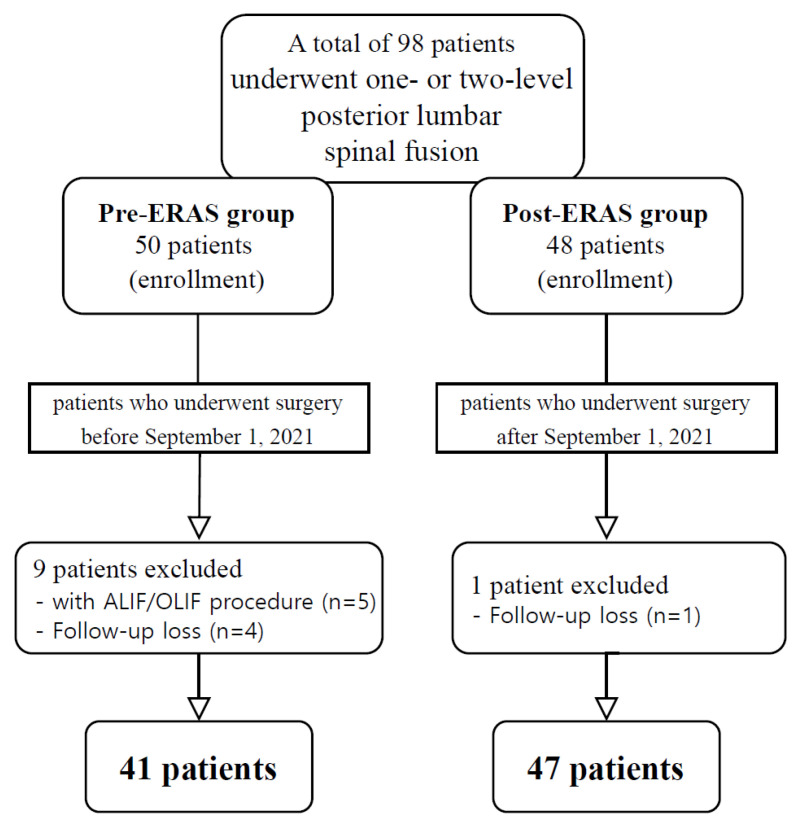
Flow diagram for the study sample selection. ERAS, Enhanced Recovery After Surgery; ALIF, anterior lumbar interbody fusion; OLIF, oblique lumbar interbody fusion.

This retrospective cohort study, conducted at the Asan Medical Center, focused on 98 patients with lumbar degenerative disease who had undergone one- or two-level posterior lumbar spinal fusion between March 2021 and February 2022. Only patients who had undergone primary surgery were included. Individuals with degenerative or isthmic spondylolisthesis, as well as those with spinal stenosis or neural foraminal stenosis requiring fusion surgery, were eligible for inclusion in this study. Patients who had undergone concomitant procedures such as anterior lumbar interbody fusion (ALIF) or oblique lumbar interbody fusion (OLIF) were excluded. Thus, 88 patients were included in the final analysis after the exclusion of 10 cases. The ERAS protocol was implemented in September 2021. The patients were allocated into two groups: those who had undergone surgery before the implementation of the ERAS protocol (pre-ERAS group, *n* = 41) and those who had undergone surgery after its implementation (post-ERAS group, *n* = 47) (Figure 1).

This study was approved by the Institutional Review Board (IRB) of our institution. All procedures were conducted in accordance with ethical standards (2022-1632). The ERAS protocol used for lumbar spinal fusion surgery at our institution is outlined in Table 1. This protocol follows the general principles of ERAS guidelines, with minor adjustments to align with our institutional practices [1,3,4,5,15].

### 2.2. Outcome Measures

The primary outcome measure of this study was pain management, as assessed using the numerical reporting scale (NRS) scores at multiple postoperative time points. The pain levels were assessed daily over the first four postoperative days (POD 1, 2, 3, and 4) using the average NRS score for each day to capture daily fluctuations in pain and provide a comprehensive evaluation of pain control during the critical early recovery period. The secondary outcome measure included the analysis of pain and functional outcomes at the 6-month postoperative time point. The NRS scores were recorded at 6 months to assess long-term pain management. The functional outcomes were evaluated using the Oswestry Disability Index (ODI) and EuroQol 5 Dimension (EQ-5D) scores recorded at 6 months postoperatively. Additional outcomes included the doses of antiemetics and opioids required, length of hospital days (HD), POD, complication rates, readmission rates within 30 days postoperatively, and fusion rates of the operated segments. The fusion rates were assessed using computed tomography (CT) at 1 year postoperatively, with fusion being defined as trabecular bridging within the cage in contact with the upper and lower endplates and the absence of a radiographic cleft [16].

### 2.3. Statistical Analysis

Descriptive statistics summarized patient demographics and baseline characteristics. Continuous variables were expressed as mean and standard deviation and categorical variables as frequencies and percentages. For the primary outcome of NRS pain scores, we used a Linear Mixed Effects Model to account for repeated measures and examine time and group effects. Separate models were fitted for back and leg pain NRS. Post hoc analyses compared group differences at each time point. Tukey-adjusted *p*-values for post hoc pairwise group comparisons at each timepoint in the Linear Mixed Effects Model were calculated to account for multiple comparisons. Secondary outcomes were analyzed using paired and independent *t*-tests for continuous variables, and Chi-square or Fisher’s exact tests for categorical variables. These tests were also used to compare antiemetic and opioid doses, hospital stay, postoperative days, complication rates, readmission rates, and fusion rates between groups. A *p*-value less than 0.05 was considered statistically significant. All analyses were performed using SPSS version 21 (IBM, Armonk, NY, USA).

## 3. Results

### 3.1. Demographics

Table 1 presents the demographic data and preoperative characteristics. No significant differences were observed between the pre-ERAS and post-ERAS groups in terms of age (*p* = 0.201); sex (*p* = 0.666) or other comorbidities, such as hypertension, diabetes, heart disease, and smoking status (Table 2).

### 3.2. Primary Outcomes

Analysis of the pain scores, measured using NRS, revealed consistent trends of lower pain scores in the post-ERAS group compared with that in the pre-ERAS group at most time points. No significant difference was observed between the two groups in terms of the pre-operative back pain scores (*p* = 0.056). The back pain scores on days 1, 2, 3, 4, and at 6 months were lower in the post-ERAS group, but these differences did not reach statistical significance (all *p* > 0.05). The most notable difference was observed on the third postoperative day (2.59 ± 0.21 vs. 2.35 ± 0.20, *p* = 0.060).

**Figure 2:**
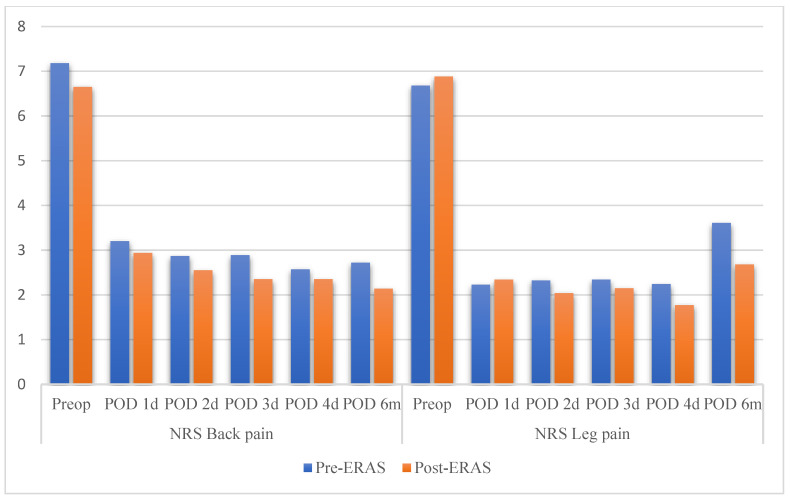
Comparison between the pre- and postoperative scores.

The pre-operative NRS scores for leg pain were comparable between the groups (*p* = 0.256). The postoperative leg pain scores in the post-ERAS group were consistently lower, but these differences were not statistically significant on days 1, 2, 3, or 4 postoperatively (all *p* > 0.05). However, a significant difference was observed at 6 months postoperatively, with the post-ERAS group reporting significantly lower leg pain (3.60 ± 0.40 vs. 2.64 ± 0.36, *p* = 0.002) (Table 3 and Figure 2).

Further analysis using the Linear Mixed Effects Model showed that the time:group interaction was not statistically significant for either back pain NRS (*p* = 0.848) and leg pain NRS (*p* = 0.503), indicating that the pattern of pain scores over time did not differ significantly between the pre-ERAS and post-ERAS groups.

### 3.3. Secondary Outcomes

Significant improvements were observed in the pre-ERAS and post-ERAS groups at 6 months postoperatively in terms of functional outcomes, as summarized in Table 4 and Table 5.

Notable improvements were observed in the following ODI domains in the pre-ERAS group: personal care (*p* < 0.001), walking (*p* = 0.016), standing (*p* = 0.005), sleeping (*p* = 0.001), social life (*p* < 0.001), and traveling (*p* = 0.001). A significant reduction in the total ODI score was observed (*p* < 0.001). Furthermore, significant improvements were observed in the following EQ-5D domains: mobility (*p* < 0.001), self-care (*p* = 0.001), usual activity (*p* < 0.001), pain/discomfort (*p* = 0.039), and anxiety/depression (*p* = 0.001).

Significant improvements were observed in the following domains in the post-ERAS group: pain (*p* < 0.001), personal care (*p* < 0.001), walking (*p* = 0.025), sitting (*p* = 0.001), standing (*p* < 0.001), sleeping (*p* = 0.020), and social life (*p* = 0.008). The total ODI score also exhibited a significant decrease (*p* = 0.003). The EQ-5D domains exhibiting significant improvements included mobility (*p* < 0.001), self-care (*p* = 0.001), usual activity (*p* = 0.002), pain/discomfort (*p* < 0.001), and anxiety/depression (*p* = 0.036).

Substantial functional recovery was observed postoperatively in both groups, with slightly greater improvements being observed in the post-ERAS group in key domains such as pain and standing, indicating the positive impact of the ERAS protocol.

### 3.4. Perioperative Medication

Analysis of perioperative medication usage revealed significant differences between the pre-ERAS and post-ERAS groups, particularly in terms of the total consumption of antiemetics and opioids.

The total usage of antiemetics in the post-ERAS group (1.41 amps) was significantly lower than that in the pre-ERAS group (2.73 amps, *p* < 0.001). No statistically significant differences were observed between the post-ERAS and pre-ERAS groups in terms of the use of individual antiemetics, such as ramosetron and palonosetron (*p* = 0.074 and *p* = 0.151, respectively); however, the overall reduction in antiemetic use in the post-ERAS group was notable.

Opioid consumption was also significantly reduced in the post-ERAS group. The mean number of amps of hydromorphone and pethidine administered in the post-ERAS and pre-ERAS groups were 1.64 and 3.13, respectively, indicating a statistically significant difference (*p* = 0.005) (Table 6).

### 3.5. Postoperative Outcomes

The comparison of postoperative outcomes between the pre-ERAS and post-ERAS groups revealed significant improvements in several key metrics following the implementation of the ERAS protocol.

#### 3.5.1. Hospital Stay and Postoperative Days

No significant differences were observed between the pre-ERAS and post-ERAS groups in terms of the average HD (9.49 days vs. 9.43 days, respectively; *p* = 0.814). Similarly, the number of POD until discharge exhibited a trend toward shorter stays in the post-ERAS group (5.23 days) compared with that in the pre-ERAS group (5.59 days); however, this difference was not statistically significant (*p* = 0.098) (Table 7).

#### 3.5.2. Complication and Re-Admission Rates

The complication rate in the post-ERAS group exhibited a significant reduction, with 4.3% (two patients) experiencing complications compared with 19.5% (eight patients) in the pre-ERAS group (*p* = 0.024). The complications observed in the pre-ERAS group included delirium (two cases), dural tear (one case), metal failure (one case), sciatica (two cases), seroma (one case), and urticaria (one case). In contrast, only one case of acute hepatitis and one case of sciatica were observed in the post-ERAS group, indicating a substantial reduction in postoperative complications following the implementation of the ERAS protocol. The re-admission rate did not differ significantly between the two groups (7.3% in pre-ERAS vs. 4.3% in post-ERAS, *p* = 0.549) (Table 7).

#### 3.5.3. Fusion Rates

The spinal fusion rates were similar in the pre-ERAS and post-ERAS groups, with fusion rates of 91.7% and 94.7%, respectively (*p* = 0.607). This finding suggests that the ERAS protocol had no adverse effect on the technical success of the spinal fusion surgery (Table 6).

## 4. Discussion

The implementation of the ERAS protocol in one- or two-level posterior lumbar spinal fusion surgeries resulted in significant improvements in postoperative outcomes, with the substantial reduction in the use of antiemetics and opioids being one of the most important findings. This reduction highlights the effectiveness of the ERAS protocol in managing postoperative symptoms; furthermore, the role of ERAS in minimizing the risks associated with opioid use and medication-related side effects is demonstrated.

The use of antiemetics in the post-ERAS group was significantly lower (*p* < 0.001), with fewer patients requiring multiple doses. To validate these findings, we conducted post hoc power analyses for our main outcomes. Our post hoc analysis revealed 99.773% power to detect the observed mean difference in antiemetic use, strongly supporting the reliability of this finding. This reduction is particularly meaningful in the context of spine surgery, wherein the incidence of postoperative nausea and vomiting (PONV) can severely delay key recovery milestones, such as early mobilization and resumption of oral intake (which are critical for enhancing surgical recovery) [17]. The incidence of PONV prolongs the recovery period and increases the likelihood of unexpected hospital admissions, contributing to higher healthcare costs and patient discomfort [18,19]. The ERAS protocol effectively minimized the requirement for the administration of antiemetics by optimizing multimodal analgesia and reducing reliance on opioids, major contributors to PONV. This facilitated faster recovery and reduced the incidence of medication-related side effects. This outcome aligns with the ERAS goals of enhancing recovery by reducing complications such as PONV and promoting early ambulation [3,4,5,12].

The reduction in opioid use is equally crucial. The opioid consumption in the post-ERAS group was significantly lower (*p* = 0.005), with our post hoc analysis showing 96.432% power to detect this difference. This finding is consistent with the core objective of ERAS, which aims to limit opioid use by incorporating the use of non-opioid analgesics such as NSAIDs, acetaminophen, and pregabalin. Effective pain control using fewer opioids decreases the risk of opioid-related complications, such as respiratory depression and gastrointestinal dysfunction. Furthermore, it also mitigates the risk of long-term opioid dependence [20]. Pain is the predominant symptom reported by adult patients undergoing ambulatory surgery and the primary cause of unplanned healthcare visits [21]. Pain levels at 48 h postoperatively are a significant predictor of the ability of a patient to resume normal activities by day 7 [22]. The patients in the post-ERAS group in this present study possibly experienced smoother recovery owing to better pain control, which contributed to earlier mobilization and fewer side effects. Moreover, the effective management of acute pain plays a crucial role in preventing the transition to chronic pain, which is typically associated with functional decline, anxiety, and depression [15,23].

No significant differences were observed between the two groups in terms of the immediate postoperative pain scores (NRS) despite these benefits. This may be attributed to the fact that a comprehensive multimodal analgesic approach was already in place before the implementation of ERAS [24,25]. The patients in the pre-ERAS group received NSAIDs, acetaminophen, pregabalin, short-term narcotics, and patient-controlled analgesia (PCA) with fentanyl and ketorolac [26]. This may have led to effective pain control in both groups, making it difficult to detect significant differences in the NRS scores. Furthermore, variability in pain assessment during the immediate postoperative period owing to patient discomfort and the fluctuation in physical conditions may have contributed to fluctuating pain reports. Measures have been taken to address this issue by calculating the daily average NRS in this present study; however, discrepancies between the pain measured at the level requiring medication after surgery and NRS measured at periodic measurement time points may have also contributed to these results.

Interestingly, while immediate postoperative pain scores showed no significant differences, our analysis revealed a significant improvement in long-term pain management, particularly for leg pain. At 6 months postoperatively, the post-ERAS group reported significantly lower leg pain scores compared to the pre-ERAS group (3.60 ± 0.40 vs. 2.64 ± 0.36, *p* = 0.002). However, our post hoc analysis showed only 25.520% power for this outcome, indicating that these results should be interpreted cautiously and larger studies are needed to confirm this finding.

To further understand the pain trajectory over time, we conducted a Linear Mixed Effects Model analysis. This analysis showed no significant time:group interaction for both back pain NRS (*p* = 0.848) and leg pain NRS (*p* = 0.503). These results indicate that the effect of the ERAS protocol remained relatively consistent over time, suggesting a sustained benefit throughout the recovery period that culminated in significantly lower leg pain at 6 months.

The discrepancy between short-term and long-term pain outcomes observed in our study is intriguing and warrants further investigation. It is possible that the cumulative effects of various ERAS components, such as early mobilization, optimized nutrition, and patient education, contribute more significantly to improved long-term pain management than to immediate postoperative pain control. This finding suggests that the ERAS protocol may have a more pronounced effect on long-term pain management rather than immediate postoperative pain control. Future studies should focus on identifying which specific elements of the ERAS protocol are most influential in promoting long-term pain reduction, while also exploring ways to enhance its impact on immediate postoperative pain management. These studies should also aim to increase statistical power, particularly for long-term pain outcomes, to provide more definitive conclusions. The postoperative pain scores remained comparable; however, the functional outcomes exhibited significant improvements in the post-ERAS group. The post-ERAS group demonstrated better scores in terms of the ODI and EQ-5D scores, particularly in domains such as pain, standing, and quality of life, at 6 months postoperatively. Significant improvements were observed in ODI domains, such as pain (*p* < 0.001), personal care (*p* < 0.001), walking (*p* = 0.025), and standing (*p* < 0.001). Consistent with the findings of earlier studies, these results indicate that the ERAS protocol can enhance therapeutic outcomes more effectively and contribute to long-term functional recovery [27].

The implementation of the ERAS protocol in our study showed promising results in terms of postoperative outcomes, including a notable reduction in complication rates from 19.5% in the pre-ERAS group to 4.3% in the post-ERAS group (*p* = 0.024). However, it is important to interpret these findings with caution due to the retrospective nature of our study and potential confounding factors.

In this retrospective study, we took several measures to minimize potential selection bias between the pre- and post-ERAS groups. We implemented strict inclusion and exclusion criteria, with all patients undergoing one- or two-level posterior lumbar fusion at a single institution. To reduce potential bias from the surgeon’s skill improvement over time, we kept the study period relatively short, from March 2021 to February 2022. We used consecutive sampling and compared baseline demographic and clinical characteristics to ensure no significant differences between groups.

Despite these efforts, the retrospective design and relatively small sample size limit our ability to draw definitive conclusions about the direct impact of ERAS on complication rates. The observed lower complication rate in the post-ERAS group might have been influenced by factors unrelated to the ERAS protocol itself, such as inherent patient variability or unaccounted perioperative care improvements. For instance, the reduced incidence of dural tears and metal failures, which are less likely to be directly influenced by ERAS protocols, suggests that the difference in complication rates may not be solely attributed to the protocol’s implementation.

Nevertheless, the significance of our findings lies in demonstrating that the adoption of ERAS protocols, which emphasize early recovery and reduced length of hospital stay, did not lead to an increase in complication rates in the post-ERAS cohort. This is a noteworthy outcome, considering that early mobilization and accelerated recovery pathways have traditionally been associated with concerns regarding increased complications. The fact that the complication rates remained low, despite the implementation of a more aggressive postoperative recovery strategy, suggests that ERAS protocols can safely enhance recovery without compromising patient safety.

These results are consistent with previous studies showing that ERAS protocols can improve recovery and minimize the need for interventions associated with adverse effects [2,12]. Our findings support the notion that ERAS protocols can be safely implemented in lumbar fusion surgeries, potentially offering the benefits of faster recovery and shorter hospital stays without increasing the risk of complications.

No significant differences in length of stay (LOS) or readmission rates were observed between the two groups in this present study. This outcome may have been influenced by institutional policies and administrative factors during hospitalization. Our institution, a tertiary hospital, had already established a target for discharge on the third postoperative day, even before the implementation of the ERAS protocol. The patients were transferred to a rehabilitation facility or discharged home depending on their condition. Nevertheless, the similar re-admission rates confirm that the ERAS protocol did not increase the risk of postoperative complications after discharge, thereby reinforcing its safety and effectiveness. Early mobilization after spinal surgery and other major procedures is associated with a shorter LOS [28,29]. Thus, encouraging earlier and more consistent ambulation in the future could further reduce the duration of hospital stay.

The fusion rates in the pre-ERAS and post-ERAS groups were also similar (*p* = 0.607), indicating that the implementation of the ERAS protocol had no negative effect on the technical success of spinal fusion surgery. This finding supports the implementation of ERAS protocols, as they enhance perioperative care without compromising the surgical outcomes.

Future studies will focus on the implementation and evaluation of the Second Generation ERAS Protocol, which incorporates several novel components designed to further enhance patient recovery, at our institution. The key features include pre-operative anemia management, reduced preoperative fasting, administration of dexamethasone on the day of the surgery and the first postoperative day, early sitting and mobilization, and early initiation of oral nutrition. These studies are underway, and the outcomes will be reported in subsequent publications.

Certain limitations of this present study must be considered. First, causality cannot be established, and unmeasured factors may have influenced the results as this was a retrospective cohort study. Furthermore, the single-institution, single-surgeon design limits the generalizability of the findings to other settings or practices. Larger multi-center studies must be conducted to enhance external validity. The sample size, although sufficient for analysis, may also limit the broader applicability of the findings. Variability in the pain scores assessed using the NRS, which relies on patient-reported outcomes, may have affected the accuracy of pain measurements, particularly during the early postoperative period [24]. Lastly, the hospital policy of discharging patients on day 3, regardless of recovery status, may have restricted the observation of potential reductions in the LOS.

Nevertheless, this present study has several strengths. The effects of the ERAS protocol could be evaluated within a well-defined surgical context by focusing on a homogeneous patient population, specifically patients undergoing one- or two-level posterior lumbar spinal fusion surgeries. This homogeneity reduced the potential effects of confounding variables, which are often observed in studies involving more complex procedures, thereby improving the internal validity of our findings. In addition, unlike those of previous studies, the objective comparison of opioid and antiemetic drug usage performed in this present study provided quantifiable metrics, adding a layer of objectivity to the analysis. A more reliable evaluation of the impact of the ERAS protocol on perioperative care could be achieved by measuring the precise dosages of these medications.

The single-surgeon and retrospective design impose some limitations; however, the focus of this present study on a specific patient group and its objective data on drug use provides valuable insights into the effectiveness of the ERAS protocols in spine surgery.

## 5. Conclusions

The implementation of the ERAS protocol in one- or two-level posterior lumbar spinal fusion surgeries demonstrated several benefits. Our study shows that this protocol significantly reduces antiemetic and opioid use while potentially improving long-term pain management and functional recovery. Importantly, these benefits were achieved without increasing complication rates, underscoring the safety of the ERAS protocol in this surgical context.

These findings contribute to the growing body of evidence supporting the effectiveness and safety of ERAS protocols in spinal surgery, while also highlighting areas for potential optimization. Our results underscore the importance of considering both short-term and long-term outcomes in evaluating these protocols, supporting their wider implementation to enhance postoperative care and patient outcomes in spinal procedures.

## Figures and Tables

**Table 1:** Comparison of Pre-ERAS and ERAS protocols in lumbar spinal fusion surgery.

Stage	Pre-ERAS Protocol	ERAS Protocol
Pre-operative Management	Fasting from midnight. No routine administration of dexamethasone, pregabalin, or celecoxib. Limited education and expectation management.	Fasting from midnight. Just before surgery: - IV administration of dexamethasone. - Oral administration of celecoxib and pregabalin. Comprehensive education.
Intraoperative Management	Less standardized pain management. No routine use of long-acting local anesthetics. Focus on general anesthesia and basic pain control.	Long-acting local anesthetics (bupivacaine) at surgical site. Tranexamic acid infusion (10 min pre-incision to wound closure). Maintain normothermia.
Postoperative Management	Opioid-based pain control (tramadol, hydromorphone, pethidine) as needed. Antiemetics and PPI administration. Delayed Foley catheter removal and ambulation. Reliance on opioids, with lesser emphasis on multimodal analgesia.	Multimodal analgesia. Scheduled medications: Acetaminophen 1 g IV post-surgery. Antiemetics. PPI. PRN medications: Tramadol, Hydromorphone, Pethidine. POD 1~: Extended-release acetaminophen, pregabalin, celecoxib, magnesium oxide, antiemetics, prokinetics. Early Foley removal. Early mobilization. Opioids as PRN.

ERAS, Enhanced Recovery After Surgery; PPI, proton pump inhibitor; PRN, Pro Re Nata (as needed); POD, postoperative day.

**Table 2:** Demographics.

	Pre-ERAS (*n* = 41)	Post-ERAS (*n* = 47)	*p*-Value
Male	14 (34.1)	14 (29.8)	0.666
Age	65.2 (±11.1)	67.8 (±8.0)	0.201
Height	158.67 (±8.06)	157.52 (±7.80)	0.501
Weight	63.98 (±12.66)	62.96 (±10.21)	0.678
BMI	25.30 (±3.81)	25.32 (±3.44)	0.980
HTN	22 (53.7)	25 (53.2)	0.966
DM	6 (14.6)	14 (29.8)	0.087
Heart disease	2 (4.9)	4 (8.5)	0.506
Liver disease	1 (2.4)	1 (2.1)	0.353
Pulmonary disease	1 (2.4)	3 (6.4)	0.381
Cancer	4 (9.8)	4 (8.5)	0.842
MDD	2 (4.9)	2 (4.3)	0.890
Smoking	5 (12.2)	5 (10.6)	0.678

ERAS, Enhanced Recovery After Surgery; HTN, hypertension; DM, diabetes mellitus; BMI, body mass index; MDD, major depressor disorder. Values are presented as number (%) of patients or mean (± SD) unless otherwise indicated.

**Table 3:** NRS in the pre-ERAS and post-ERAS groups.

		Pre-ERAS	Post-ERAS	*p* Value
NRS Back pain	Preop	7.20 (±0.22)	6.63 (±0.20)	0.056
	Postop#1D	3.20 (±0.21)	2.93 (±0.20)	0.358
	Postop#2D	2.87 (±0.21)	2.25 (±0.20)	0.266
	Postop#3D	2.59 (±0.21)	2.35 (±0.20)	0.060
	Postop#4D	2.57 (±0.21)	2.36 (±0.20)	0.446
	Postop#6M	2.70 (±0.30)	2.06 (±0.28)	0.119
NRS Leg pain	Preop	6.68 (±0.27)	6.88 (±0.26)	0.256
	Postop#1D	2.19 (±0.31)	2.33 (±0.30)	0.661
	Postop#2D	2.29 (±0.31)	2.03 (±0.31)	0.301
	Postop#3D	2.31 (±0.30)	2.14 (±0.30)	0.496
	Postop#4D	2.22 (±0.30)	1.77 (±0.30)	0.404
	Postop#6M	3.60 (±0.40) *	2.64 (±0.36) *	0.002

NRS, numerical reporting scale; Preop, Preoperative; Postop, Postoperative. Values are presented as mean (95% CI) unless otherwise indicated. * *p* < 0.005.

**Table 4:** ODI and EQ-5D scores in the pre-ERAS group.

ODI	Pre-Operative	Postoperative 6 m	*p*-Value
Pain	3.25 (±0.775)	2.38 (±1.746)	0.084
Personal care	2.00 (±0.632) *	0.69 (±0.793) *	0.000
Lifting	3.25 (±1.065)	3.44 (±1.209)	0.676
Walking	2.57 (±1.284) *	1.07 (±1.439) *	0.016
Sitting	2.50 (±1.095)	1.69 (±1.352)	0.055
Standing	3.56 (±1.153) *	2.06 (±1.526) *	0.005
Sleeping	2.50 (±1.414) *	0.63 (±1.025) *	0.001
Social life	2.94 (±0.854) *	1.50 (±1.211) *	0.000
Traveling	3.00 (±1.195) *	1.07 (±1.223) *	0.001
Sex life	-	-	
Total	27.00 (±7.598) *	14.50 (±7.755) *	0.000
**EQ-5D**			
Mobility	3.44 (±0.814) *	1.81 (±0.834) *	0.000
Self-care	2.19 (±0.750) *	1.38 (±0.619) *	0.001
Usual activity	3.19 (±0.911) *	1.69 (±0.704) *	0.000
Pain discomfort	3.88 (±0.719) *	3.00 (±1.265) *	0.039
Anxiety depression	2.25 (±1.000) *	1.38 (±0.619) *	0.001

ODI, Oswestry Disability Index; EQ-5D, EuroQol 5 Dimension (EQ-5D) questionnaire. Values are presented as mean (± SD) unless otherwise indicated. * *p* < 0.005.

**Table 5:** ODI and EQ-5D scores in the post-ERAS group.

ODI	Pre-Operative	Postoperative 6 m	*p*-Value
Pain	3.35 (±1.115) *	1.41 (±1.004) *	0.000
Personal care	2.35 (±1.412) *	0.47 (±0.624) *	0.000
Lifting	3.35 (±1.272)	3.00 (±1.803)	0.524
Walking	2.59 (±1.417) *	1.47 (±1.663) *	0.025
Sitting	2.65 (±1.057) *	1.29 (±1.404) *	0.001
Standing	3.35 (±1.367) *	1.29 (±1.448) *	0.000
Sleeping	1.65 (±1.169) *	0.76 (±0.903) *	0.020
Social life	2.82 (±1.185) *	1.41 (±1.661) *	0.008
Traveling	2.35 (±1.539)	2.12 (±2.118)	0.660
Sex life	4.00 (±0.000)	4.00 (±0.000)	1.000
Total	27.33 (±7.898) *	15.27 (±9.098) *	0.003
**EQ-5D**			
Mobility	3.44 (±1.094) *	1.94 (±0.854) *	0.000
Self-care	2.38 (±1.025) *	1.19 (±0.403) *	0.001
Usual activity	2.63 (±0.806) *	1.75 (±0.683) *	0.002
Pain discomfort	3.75 (±0.931) *	2.13 (±0.719) *	0.000
Anxiety depression	2.44 (±1.153) *	1.75 (±1.000) *	0.036

ODI, Oswestry Disability Index; EQ-5D, EuroQol 5 Dimension (EQ-5D) questionnaire. Values are presented as mean (± SD) unless otherwise indicated. * *p* < 0.005.

**Table 6:** Perioperative medication.

Antiemetic Drug	Pre-ERAS		Post-ERAS		*p*-Value
	Patient Number	Ampule	Patient Number	Ampule	
1. Ramosetron	41	2.32 (±0.82)	8	1.75 (±0.71)	0.074
2. Palonosetron	10	1.50 (±0.71)	37	1.14 (±0.48)	0.151
3. Macperan	2	1.00 (±0.00)	2	2.50 (±0.71)	0.095
4. Onseran	0	.	1	1.00 (±0.00)	
Total	41	2.73 (±1.34) *	44	1.41 (±1.15) *	<0.001
**Opioid**	**Pre-ERAS**		**Post-ERAS**		***p*-value**
	**Patient number**	**Ampule**	**Patient number**	**Ampule**	
Hydromorphone and Pethidine	23	3.13 * (±2.32)	14	1.64 * (±0.93)	0.005

ERAS, Enhanced Recovery After Surgery. Values are presented as mean (± SD) unless otherwise indicated. * *p* < 0.005.

**Table 7:** Comparison between the postoperative data.

	Pre-ERAS	Post-ERAS	*p*-Value
HD	9.49 (±1.33)	9.43 (±1.16)	0.814
POD	5.59 (±1.07)	5.23 (±0.87)	0.098
Re-admission rate	7.3%	4.3%	0.549
Complication rate	19.5% *	4.3% *	0.024
Fusion rate	91.7%	94.7%	0.607

ERAS, Enhanced Recovery After Surgery; HD, Hospital day; POD, Postoperative day. Values are presented as mean (95% CI) unless otherwise indicated. * *p* < 0.005.

## Data Availability

The data supporting the findings of this study are not publicly available due to privacy and ethical restrictions. However, de-identified datasets are available from the corresponding author upon reasonable request, subject to approval by the Institutional Review Board of Asan Medical Center. The data will be shared in accordance with the principles of FAIR (Findable, Accessible, Interoperable, and Reusable) data practices while ensuring compliance with ethical guidelines and participant confidentiality.
